# Use of Optical Coherence Tomography in Dentistry

**DOI:** 10.1155/2023/4179210

**Published:** 2023-12-11

**Authors:** Omer Sefvan Janjua, Waqar Jeelani, Muhammad Ikram Khan, Sana Mehmood Qureshi, Muhammad Saad Shaikh, Muhammad Sohail Zafar, Zohaib Khurshid

**Affiliations:** ^1^Department of Oral and Maxillofacial Surgery, PMC Dental Institute, Faisalabad Medical University, Faisalabad, Pakistan; ^2^Department of Orthodontics, College of Dentistry, Bakhtawar Amin Medical and Dental College, Multan, Pakistan; ^3^Dentist private practice, Sydney, NSW, Australia; ^4^Department of Oral Pathology, PMC Dental Institute, Faisalabad Medical University, Faisalabad, Pakistan; ^5^Department of Oral Biology, Sindh Institute of Oral Health Sciences, Jinnah Sindh Medical University, Karachi 75510, Pakistan; ^6^Department of Restorative Dentistry, College of Dentistry, Taibah University, Al Madinah, Al Munawwarah, 41311, Saudi Arabia; ^7^School of Dentistry, University of Jordan, Amman 11942, Jordan; ^8^Department of Dental Materials, Islamic International Dental College, Riphah International University, Islamabad 44000, Pakistan; ^9^Department of Prosthodontics and Dental Implantology, College of Dentistry, King Faisal University, Al-Ahsa 31982, Saudi Arabia

## Abstract

Optical coherence tomography (OCT) is an optics-based imaging technique, which may be called an “optical biopsy.” It can be used to acquire structural information about a tissue at a resolution comparable to histopathology. OCT is based on the principle of low-coherence interferometry where near-infrared (NIR) light is shown on a tissue sample and then cross-sectional images are obtained based on backscattered light and echo time delay. Two main types of OCT are characterized as time-domain OCT (TD-OCT) and Fourier-domain OCT (FD-OCT). The applications of OCT in dentistry can be broadly divided into two groups, i.e., assessment of pathologies and assessment of surfaces and interfaces. Lately, OCT has made its transition from experimental laboratories to mainstream clinical applications. Starting from the short-term training courses, clinicians working in specialities like oral pathology, oral medicine, and oral implantology may find it a useful tool for their practices. It is now clear that OCT will be considered a gold standard diagnostic tool for the detection and characterization of several conditions and lesions of the orofacial region. However, the next challenge will be to incorporate it into the undergraduate and postgraduate curriculum and train dental healthcare staff in the use of these devices.

## 1. Introduction

Diagnostic imaging is considered the backbone of medicine and dentistry. The development of noninvasive diagnostic modalities with minimal or no side effects has been a top priority for the last few decades. Since the dawn of the 21st century, there has been a paradigm shift from two-dimensional imaging to three-dimensional (3D) imaging [cone beam computed tomography (CBCT) imaging, 3D ultrasound, 3D photography, etc.] [1]. The possibility of using ultrasounds for the analysis of superficial and non-surface structures by modifying the ultrasound frequency inside the tissues and through the use of probes indicated for oral application is important yet underestimated, a factor which until now has prevented the spread of the use of this technology in dentistry, as indicated by Elbarbary et al. [2]. In a fashion similar to ultrasound, which utilizes high-frequency sound waves and CT scans, which employ X-rays, optical coherence tomography (OCT) makes use of optics or a light-based system, which renders a 3D image of the underlying tissues. In terms of image resolution and depth of penetration in the tissues, OCT lies between optical microscopy and ultrasound (US) imaging [3].

A team at the Massachusetts Institute of Technology floated the concept of OCT in 1991 and initially, it was marketed for nondestructive industrial testing and ophthalmological applications in the field of medicine [4]. However, soon it was taken up by cardiologists, gastroenterologists, and dermatologists to be used for diagnostic purposes. The current literature describes a wide spectrum of applications of OCT in the dental field, especially for the diagnosis of caries and oral mucosal lesions [5].

OCT is an optics-based imaging technique, which may be called an “optical biopsy” and can be used to acquire structural information about a tissue at a resolution comparable to histopathology without the need to extract the sample from the body [3]. Principally, OCT is based on the concept of low-coherence interferometry where near-infrared (NIR) light is shown on a tissue sample and then cross-sectional images are obtained based on backscattered light and echo time delay, which is analyzed by a detector and an image is produced on the screen. The latest research is focused on making OCT a high-speed imaging modality that allows accurate and rapid data acquisition. The second challenge of bulky equipment and difficult access to the closed cavities has also been overcome and now both the detector and the source of OCT have been significantly improved [6]. This has led to the development of handheld devices, which can be integrated into endoscopes, catheters, and biopsy needles, significantly widening the horizon of applications and uses in clinical healthcare [7].  Table 1 shows the properties of OCT with other imaging techniques used for similar purposes.

The field of OCT has seen rapid transition from bench to bedside and now finds wide applications in the field of healthcare and specifically dentistry. The key features of OCT that make it an attractive diagnostic tool include rendering an image with 0.5 *μ*m resolution, use of harmless light source, fiber-optic-based systems making it handy and pliable, ability to use inside body cavities and blood vessels, and ability to measure flow and spatial structural arrangement of the tissues [8, 9].

A survey of the published literature showed only a few in-depth reviews about the use of OCT in dentistry. This paper aims to review the principles of OCT, its types, and the range of clinical and laboratory applications in dentistry. This paper also outlines the limitations of OCT in relevance to dentistry.

OCT and its application in clinical dentistry is a relatively novel and emerging topic with new data appearing every day. Following the Scale for the Assessment of Narrative Review Articles (SANRA) guidelines [10], the objective of this paper was to comprehensively review the available data on the techniques and application of OCT in various disciplines of dentistry and in order to achieve this objective, the following questions were formulated prior to conducting the literature review: (i) What is OCT? (ii) What are the different techniques and types of OCT? (iii) What are the overall advantages of OCT over other available diagnostic modalities? (iv) What are the different applications and potential roles of OCT in dentistry? (v) What are the limitations and drawbacks of OCT, which restrict its routine use? With these questions in mind, literature was searched for abstracts and full-length papers on the subject of OCT in dentistry using key terms like OPTICAL COHERENCE TOMOGRAPHY, LIMITATIONS, FOURIER AND TIME-DOMAIN OCT, HANDHELD OCT DEVICES, OCT and RESTORATIVE DENTISTRY, IMPLANT DENTISTRY and OCT, ORAL PATHOLOGICAL LESIONS, PROSTHODONTICS, NCCLS, ENDODONTICS, GINGIVAL REGENERATION, POCKET DEPTH ASSESSMENT, LIMITATIONS, PITFALLS, etc. Therefore, this review is an attempt to provide scientific data and reasoning on the subject of OCT in different domains and disciplines of dentistry.

## 2. Types of OCT

The two major types of OCT are categorized as time-domain OCT (TD-OCT) and Fourier-domain OCT (FD-OCT).

### 2.1. Time-Domain OCT (TD-OCT)

**Figure 1:**
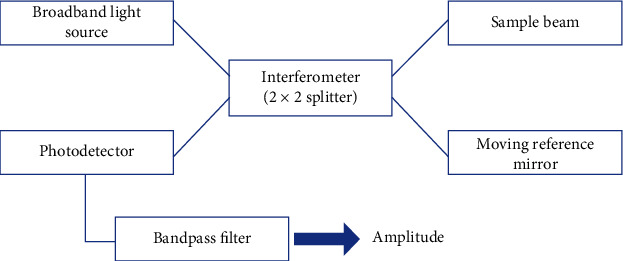
Schematic representation of time-domain OCT.

This was the originally conceived idea and is based on the concept of white light interferometry. It utilizes a broadband light source and a Michelson/Mach–Zehnder interferometer [11]. A beam splitter splits the low coherent light coming from the source, which is a superluminescent diode (SLD) and one arm of the split beam goes to a reference mirror and the other toward the sample/biological tissue. Upon its reflection from the tissues, referred to as backscattering, the light is detected by a photodetector, which compares the backscattered light from the tissue/sample with the reference arm and generates an image. In TD-OCT, the reference arm and the tissue probe both move and it is the movement of the reference mirror, which limits the imaging speed and makes the equipment cumbersome and less suitable for most of the clinical applications. The image resolution achieved with TD-OCT is 10 *µ*m or less.  Figure 1 shows schematic representation of TD-OCT.

### 2.2. Fourier-Domain OCT

FD-OCT was developed as a refinement of TD-OCT and does not contain a moving reference arm, which leads to better imaging speeds and reduces the scanning delay. FD-OCT has two further subcategories, i.e., spectral domain OCT (SD-OCT) and the swept-source OCT (SS-OCT).

The SD-OCT uses a spectrometer instead of a photodetector for image acquisition. Here, the spectrometer detects the whole optical spectra of the light, thus utilizing all the wavelengths of the light to provide information about the sample/tissue under analysis. A Fourier transform is then used to produce image.

**Figure 2:**
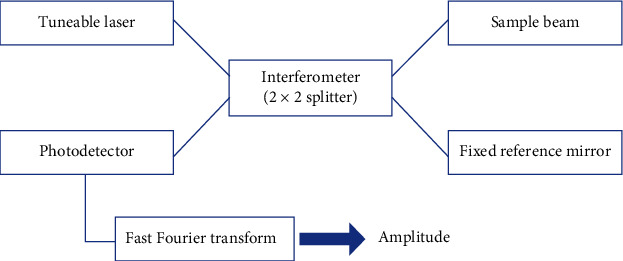
Schematic representation of Fourier domain OCT.

**Figure 3:**
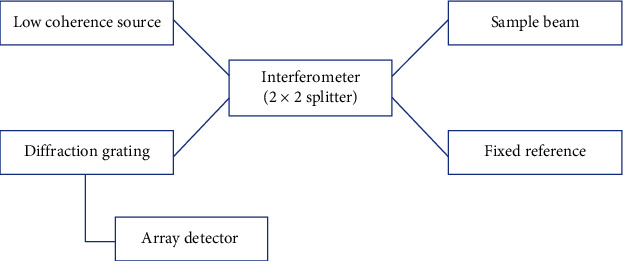
Schematic representation of spectral domain OCT.

**Figure 4:**
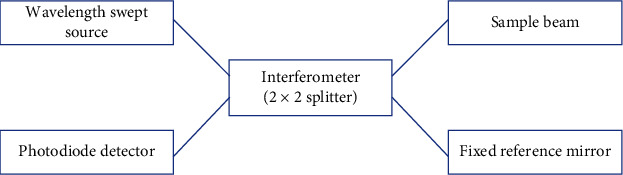
Schematic representation of swept-source OCT.

The SS-OCT utilizes a wavelength-swept laser instead of SLD and a photodetector. The final image is obtained through Fourier transform and is based on frequency-dependent light deflection. It has a well-established benefit over TD-OCT in terms of signal-to-noise ratio and data acquisition speed with the resolution dependent upon the light source bandwidth. The average bandwidth is around 100 nm and a resolution of 7.4 *µ*m. Figures 2–4 show schematic representation of FD-OCT, SD-OCT, and SS-OCT, respectively.

### 2.3. Polarization-Sensitive and Cross-Polarized OCT

A rapid 2D/3D imaging of either transparent or translucent samples can be performed using polarization-sensitive OCT (PS-OCT) using micrometer scale resolution. Tissue-specific contrast is generated when changes in the state of polarization are detected by PS-OCT due to change in the probing light [12, 13].

Cross-polarization OCT (CP-OCT) is another variant of PS-OCT. It has been ability to detect alterations in the initial polarization due to birefringence and cross-scattering and, thus, can form an image [14]. In CP-OCT, two images, i.e., parallel (which is also referred to as a conventional OCT image or image in copolarization) and orthogonal (image in cross-polarization) are coregistered to obtain a visual representation of the properties of the tissue being studied [14].

## 3. Handheld/Mobile Devices for OCT

The introduction of handheld or mobile devices for OCT has played a key role in incorporating this new diagnostic modality in various specialities such as otorhinolaryngology, ophthalmology, and dentistry. The size, design, and grip are now being customized as per the demands of various specialties. The tube-like device systems with endoscope heads are preferred by dentists, while the pistol-like grip is used by an ophthalmologist. Moreover, depending on the diagnostic needs and access to the site of interest, various types of scanning heads and adapters are now available, making it a practical option for several purposes [6].

## 4. Clinical Applications

**Figure 5:**
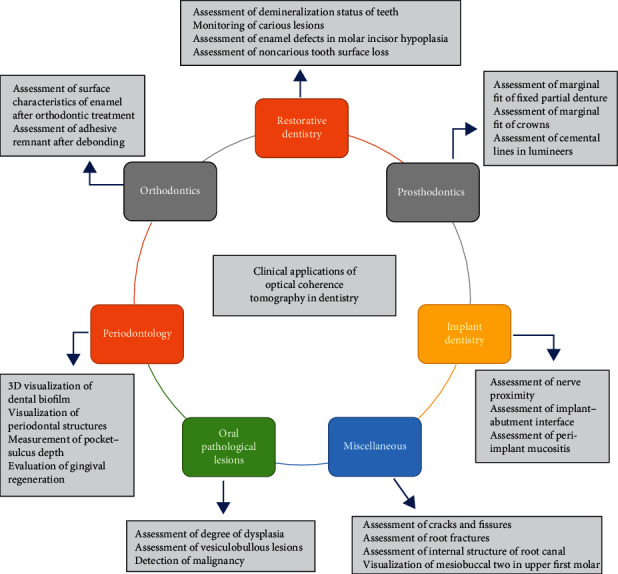
Flow diagram showing applications of OCT in dentistry.

Figure 5 demonstrates various clinical applications of OCT in different dental specialties.

### 4.1. Restorative Dentistry/Cariology

Demineralization of enamel and dentin is one of the most common problems encountered in dentistry. Early detection and treatment of enamel and dentin caries significantly reduce the extent and cost of treatment for the patient, as preventive regimens can be used, which can help avoid extensive restorative treatments. OCT finds its major applications in the discipline of restorative dentistry especially in the field of cariology and caries detection.

#### 4.1.1. Assessment of Demineralization Status of Teeth

In routine dentistry, detection of early carious lesions relies on clinical examination and bitewing radiographs that expose the patient and the staff to ionizing radiation. Detection of proximal lesions is particularly challenging, as the field is obscured. Similarly, the evaluation of remaining dentin thickness (RDT) in deep carious lesions is also unreliable on 2D radiographic images [15].

**Figure 6:**
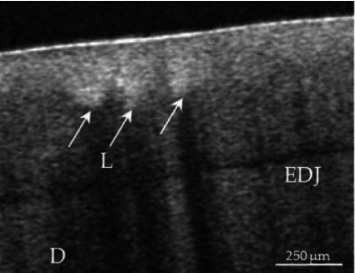
The image showing OCT image of an incipient carious lesion in enamel (white arrows) [16].

Early detection of caries lesions is possible using OCT without exposing patient or staff to any type of ionizing radiation [16]. Moreover, it can also be used for the purpose of monitoring of early progression of dental caries and the robustness of restorative treatment carried out for the management of caries at minimal costs over a long period of time (Figure 6). This can provide quick and clinically relevant information at a minimal cost [17].

#### 4.1.2. Monitoring of Caries after Restoration/Fluoride Treatment

**Figure 7:**
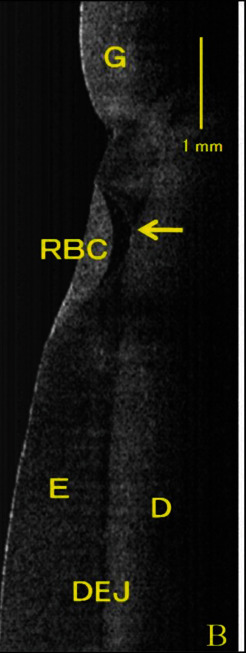
An OCT image showing cervical lesion restored with composite. Arrow points to microleakage under the restoration. G, gingiva; RBC, resin-based composite; E, enamel; D, dentin; DEJ, dentinoenamel junction [29].

Tom et al. [18] showed that the customized OCT with a wavelength region between 1500 and 1700 nm can generate the highest contrast of lesions under sealants. NIR OCT reflectance and transillumination change significantly with respect to the adjacent sound enamel in cases of increased water absorption. This can be used to detect marginal leakage after restorations (Figure 7).

Fluoride treatment is one of the primary modalities used for both preventive and therapeutic purposes. CP-OCT has been used to detect the changes in enamel and early carious lesions after the application of fluoride varnish. The findings of Chan et al. [19] recommend the use of CP-OCT for the evaluation and monitoring of enamel surface zone that is the primary area of interest in cases of early carious lesions or fluoride treatment.

#### 4.1.3. Assessment of Enamel Defects in Molar Incisor Hypoplasia (MIH) Cases

Molar incisor hypoplasia (MIH) is a common condition affecting about 14% of global population [20]. OCT imaging can be beneficial in identifying the type and extent of enamel defects in MIH with an additional benefit of being a radiation-free diagnostic modality [21].

#### 4.1.4. Assessment of Noncarious Tooth Surface Loss (NCTSL)

As the name indicates, noncarious tooth surface loss (NCTSL) is loss of dental tissue from causes other than caries and generally includes erosion from intrinsic or extrinsic acids, attrition from physical wear, abrasion through tooth brushing, etc., and abfraction, which is flexural damage to the tooth structure due to increased occlusal loading [22]. The extent of hard tissue damage in these cases depends on intensity, frequency, duration, and magnitude of the forces.

NCTSL is a usually a slow process (average loss around 30–55 *µ*m/year in normal individuals and can be as high as 1 mm/year in severe disease) and requires long-term monitoring, which includes regular clinical and radiological examinations [23]. These serial examinations include various indices and use of study casts to determine the progression/stability of the tooth wear process. Smith and Knight [24] developed a tooth wear index (TWI), which is regularly employed in clinical setups but is not 100% accurate.

MărcăuĠeanu et al. [25] demonstrated OCT to have the potential for non-invasive early detection and long-term monitoring of NCTSL. They were able to show microstructural patterns in erosion, attrition, and abfraction using C-scans and B-scans.

### 4.2. Orthodontics

Comprehensive orthodontic treatment usually lasts for several months that jeopardize the oral health by making the maintenance of oral hygiene challenging. The enamel demineralization is one of the most common complications of fixed orthodontic treatment. Second, the process of debonding may also damage enamel surface producing enamel cracks or reducing the overall thickness of enamel.

#### 4.2.1. Characteristics of Surface Enamel after Orthodontic Treatment

Koprowski et al. [26] proposed a method for automatic quantitative assessment of enamel thickness using the OCT scans. This method allows effective cross-sectional evaluation of surface enamel after fixed orthodontic treatment. A reduction in the thickness of enamel after debonding has been observed irrespective of the type of adhesive system used. This reduction in enamel surface after completion of orthodontic treatment can be effectively assessed through the use of OCT and can be compared with pretreatment values [27]. Similarly, Nee et al. [28] demonstrated the ability of CP-OCT to measure a significantly increased demineralization in the region of orthodontic brackets over a year.

#### 4.2.2. To Assess Remnants of Adhesive Materials after Debonding

The evaluation of adhesive remnant on the tooth surface after debonding can be effectively performed utilizing OCT-based 2D/3D images [28–30]. OCT can, thus, act as an important diagnostic tool for the evaluation of debonding procedures both in clinical and research settings.

### 4.3. Periodontology

Gingival and periodontal diseases are mostly attributed to plaque, which is a bacterial biofilm forming on the tooth surface and is referred to as calculus once mineralized. In routine dental practice, plaque is evaluated through clinical examination or by using disclosing solutions or tablets. Won et al. [7] found out in a recent study about the limitations of customized handheld OCT devices in clinical assessment of gingiva and dental plaque.

PS-OCT has the ability to differentiate between enamel and the dental biofilm on the basis of birefringence of the hydroxyapatite crystals in enamel. In a clinical setting, the handheld OCT imaging system can be utilized effectively to gather depth-resolved information about gingiva and dental biofilm [7].

#### 4.3.1. Visualization of Periodontal Structures

**Figure 8:**
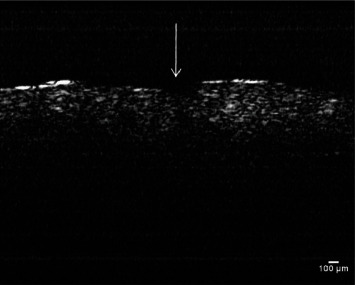
OCT image showing ulcerative lesion (arrowed) in the gingival epithelium of a periodontal disease patient [39].

OCT has the ability to evaluate periodontal tissues in vivo and can function as a reliable tool for sulcus depth measurements in healthy as well as diseased sites (Figure 8). This utilization is somewhat limited to the anterior regions of the mouth due to the inaccessibility of posterior regions by the OCT probe and further technological refinements are still needed to improve the evaluation of posterior oral sites through OCT [7].

Clinical attachment loss can also be adequately quantified using OCT. The mechanical scanning speed for image acquisition limits the conventional TD-OCT system; in this regard, therefore, Damodaran et al. [31] have introduced a novel electro-optic-based scanning system, which has the potential to overcome the speed issue of TD-OCT system.

#### 4.3.2. 3D Characterization of Biofilm

Assessment of biofilm growth over tooth surface or restorations helps in the identification of type and severity of periodontal disease. This biofilm is destroyed during routine dental prophylaxis. CP-OCT can give a 3D characterization of the biofilm based on depth-resolved scattering and elucidate the growth of certain microorganisms in the intraoral environment in a non-destructive way [32, 33].

Various antibiofilm agents are used to decrease intraoral bacterial counts. The rapid interactions between these antibiofilm agents and the hard and soft tissue surfaces of the oral cavity can be examined using a novel CP-OCT flow cell assay [34].

#### 4.3.3. Measurement of Sulcus/Pocket Depth

Pocket depth evaluation and measurement of clinical attachment loss can be done adequately through OCT. Accurate axial resolution is determined by calculating the calibration factor of OCT, which can be employed to measure periodontal pocket depth quantitatively [35].

The effectiveness of an OCT system to record the pocket depth accurately depends upon the penetrability of the NIR electromagnetic waves. Generally, the systems operating at a longer wavelength show improved accuracy, as a longer central wavelength can penetrate tissues deeper [36].

Park et al. [37] compared the diagnostic accuracy of OCT with that of micro-CT and histology in periodontal disease states. The results of their study showed that OCT has the potential to correctly identify periodontal tissue contour, sulcus depth, and the presence of supra- and sub-gingival calculus. They stated that the image depth was limited to 1.2–1.5 mm but within this depth, the surface characteristics of the dentogingival complex were picked up better by OCT as compared to micro-CT but less optimal than histological measurements.

#### 4.3.4. Evaluation of Gingival Regeneration

After gingival surgical procedure, the epithelium and connective tissue undergo a series of events to heal completely. Different stages of healing give different appearances at a microscopic level. OCT has been shown to be an effective and non-invasive method in the evaluation of regeneration of gingival tissue after esthetic periodontal surgery [38].

### 4.4. Prosthodontics

The precise fit of any fixed partial denture is paramount for its success. Failure to achieve this fit may result in marginal leakage and ultimately the failure of the prosthesis. Park et al. [40] have used a chairside optical scanner to assess the fit of a fixed partial denture. This in vivo method gives 3D digital analysis in simple three steps. In the first step, an extraoral image of the actual restoration is taken, the second step is the acquisition of an intraoral image of the abutment tooth, and in the last step, an intraoral image is taken after seating the restoration on the abutment tooth.

Similarly, the cemental lines in lumineers not only define its long-term success but also the esthetic outcome. Fernandes et al. [41] demonstrated the promise and effectiveness of OCT in the clinical evaluation of the cementing lines in lumineers.

Last, the marginal seat of crown restorations can also be assessed by OCT. Early diagnosis of faults associated with the ceramic crowns is now possible prior to inserting in the mouth, thereby reducing the overall risk of failure of a prosthetic treatment [42]. According to Gabor et al. [43], OCT-based evaluation of all ceramic crowns, which have been fabricated through digital impressions, and the CAD/CAM technology shows a superior result as compared to the conventional impression technique.

### 4.5. Oral Pathological Lesions

OCT finds its application in oral pathological lesions where collagen is being lost or remodeled. At sites of active inflammation, there is disorganization of collagen fibers, which leads to attenuation of OCT. Similarly, there is OCT signal amplification in areas where new collagen is being formed. The resultant image can be interpreted accordingly and can be utilized in the assessment of dysplasia, vesiculobullous lesions, and malignancy cases [44].

#### 4.5.1. Degree of Dysplasia

Different OCT systems working with different NIR wavelengths have been helpful in evaluating the most representative site of the biopsy. The 890 nm wavelength OCT provides the advantage of a high degree of contrast between normal and dysplastic tissue. This ability to differentiate between normal and precancerous/cancerous tissue is further amplified at 1300 nm wavelength, as it can penetrate better through the keratinized layer [45]. This quantitative differentiation between normal/abnormal sites using OCT offers a noninvasive objective approach. Thus, OCT can act as screening tool for oral cancer and precancer and, second, it can help in identifying the most representative site for performing a biopsy [46].

#### 4.5.2. Detection of Malignancy

The limited depth of penetration of OCT restricts its use in bone-related pathologies; however, further research is underway for the possibility of this application in bone-related pathologies. Future research should focus on a wavelength, which can penetrate hard tissue more successfully. Faster and more efficient OCT systems possessing higher resolution may replace the need for biopsies in many clinical situations in the future [47].

#### 4.5.3. Vesiculobullous Lesions

The ability of OCT to pinpoint epithelial changes describes its ability to give accurate picture of various vesiculobullous and vascular lesions. Adegun et al. [48] have shown that OCT possesses the ability to differentiate normal mucosa from fluid-filled areas with a sensitivity and specificity of around 80%. OCT can be a potential clinical tool that can allow non-invasive objective visualization of the oral mucosa in vesiculobullous lesions.

### 4.6. Implant Dentistry

Placement of a successful dental implant depends on several factors. Among biological factors, the proximity of implant to the neurovascular bundle, the fit at implant–abutment interface, and peri-implant mucositis are few things that can be accurately assessed using OCT systems.

#### 4.6.1. Assessment of Neurovascular Bundle Proximity

One of the major concerns while placing a dental implant in the posterior mandible is an assessment of proximity of the implant's apex to the inferior alveolar canal. OCT has been shown to be valuable in tracking the proximity of the neurovascular bundle to the implant in real-time. The accuracy of OCT in this regard is in the range of a few millimeters when using NIR signals. This accuracy is further enhanced to submillimeter range when an OCT device with a higher resolution is utilized [49].

#### 4.6.2. Implant–Abutment Interface

Improper fit at implant–abutment interface and cement remnants at the submucosal areas are common reasons for post-restorative complications in implant-supported prosthesis. OCT can effectively guide the clinician in the evaluation of the inappropriateness of implant–abutment interface under the gingival soft tissue [50]. Remnants of residual cement in the submucosa can also be picked up by OCT image at the time of prosthesis cementation, which can be beneficial in preventing residual cement-related peri-implant disease later on.

#### 4.6.3. Assessment of Peri-Implant Mucositis

The potential of an OCT device to give a detailed picture of epithelial and connective tissue structures has found its place in implant dentistry in the assessment of peri-implant mucositis as well. Clinical applications of OCT imaging are being developed for early detection of peri-implant mucositis, which, in turn, could lead to early therapeutic interventions in order to reduce the chances of implant failure due to peri-implant mucositis [51].

### 4.7. Miscellaneous Applications of OCT in Dentistry

**Figure 9:**
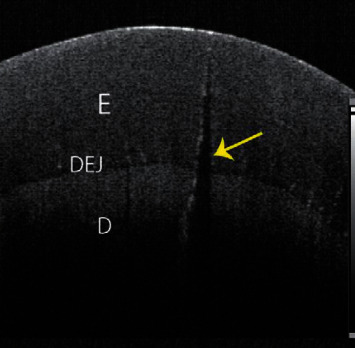
An OCT image showing crack (arrowed) propagating from dentin toward enamel crossing the DEJ [26].

Cracks and fractures of enamel and dentin are associated with several symptoms ranging from mild hypersensitivity to tooth devitalization. Cracks and fractures are routinely diagnosed through clinical examination coupled with transillumination, which aids in identifying the depth and severity of the crack [52]. Fried et al. [53] used NIR OCT imaging at 1300 nm wavelength for in vitro and in vivo assessment and identification of cracks in enamel and dentin (Figure 9). They used SS-OCT images, found crack lines to possess distinct characteristics, and identified structural crack and craze lines through SS-OCT images. Therefore, SS-OCT is considered a reliable diagnostic tool for the diagnosis of cracked tooth syndrome. Segarra et al. [54] found a close relationship between crack pattern, type of tooth, and crack's location on the tooth. Their work demonstrated that 3D SS-OCT allows for nondestructive 3D imaging and analysis of cracked enamel. Last, the use of 3D OCT has been demonstrated to be an effective tool for the identification of root fractures [55]. In this context, an in vivo study reports OCT to be superior to the digital radiography and CBCT when it comes to detecting vertical root fractures [56].

Assessment of root canal system is paramount for successful root canal therapy and post and core buildups. An ex vivo study pointed out that the internal root morphology could be seen by OCT and the image becomes further clear if the cementum is removed [57]. The OCT images give better results as compared to micro-CT scan images for identifying gaps at the tooth and post/core interface. Another study reports that real-time OCT using continuous tomographic images allows successful construction of a video of the resin core buildup, thus helping clinicians to achieve better seal at the tooth and post margins [58].

Identifying the second mesiobuccal canal (MB2) is often challenging. SS-OCT imaging has been shown to be helpful for the detection of MB2 in maxillary first molars [59]. All these applications demonstrate the potential that OCT has to transform the day-to-day dental practice.

## 5. Future

The deal with the primary drawback of limited penetration of NIR waves, newer OCT systems incorporating longer wavelengths >1300 nm are likely to give a bigger picture rather than only surface characteristics. The second challenge of bulky and expensive units is likely to be resolved very soon. In the era of innovation in technology, we have already started to witness specialty-specific and site-specific OCT systems. There are reports of several handheld systems with miniature tips for intraoral use for dental application.

As more data are available from in vivo studies, the OCT will find its place in the day-to-day dental practice. In future, OCT may be considered as a gold standard diagnostic tool for the detection and characterization of several conditions and lesions of the orofacial region. However, the next biggest challenge will be to incorporate it into the undergraduate and postgraduate curriculum and train dental healthcare staff in the use of these devices.

## 6. Conclusion

OCT uses optics- or light-based system that renders a 3D image of the underlying tissues. Over the last few years, OCT has made its transition from experimental laboratories to mainstream clinical applications. We have already started to witness the specialty-specific and site-specific OCT systems. There are reports of several handheld systems with miniature tips for intraoral use for dental application. Starting from the short-term training courses, clinicians working in specialties like oral pathology, oral medicine, and oral implantology may find it a useful tool for their practices. However, their mainstream utility will be for research fellows working to improve the quality of dental materials and clinical care. In future, OCT may be considered as a gold standard diagnostic tool for the detection and characterization of several conditions and lesions of the orofacial region.

## Figures and Tables

**Table 1:** Showing comparison of OCT with other imaging modalities.

	OCT	Ultrasound	MRI	Fluoroscopy	Angioscopy
Resolution (*µ*m)	1–15	80–120	80–300	100–200	<200
Probe size (*µ*m)	140	700	N/A	N/A	800
Ionizing radiation	No	No	No	Yes	No

MRI, magnetic resonance imaging; OCT, optical coherence tomography.

## Additional Points

*Limitations*. One of the biggest limitations is the limited depth of penetration in the tissues by the near-infrared light (currently limited to 1–3 mm). This limits the use of analysis of thin tissues such as epithelium, surface enamel, or biofilms for the time being. A high-quality light source with improved tissue penetration can overcome this limitation; however, the cost of the equipment becomes significant. In dentistry, usual measurements are in centimeters and in order to capture a lesion of that size completely, an operator has to take multiple images. This is tedious, time-consuming, and uncomfortable for the operator and the patient. The quality of individual teeth varies between individuals and even within the oral cavity of the same individual. The relative thickness of enamel and dentin and/or the presence of restorative materials alter the absorption of light and this can adversely affect the quality of the OCT image. This image distortion hampers correct interpretation [60]. Rough, irregular, and nonhomogeneous tissue composition affects the penetration and depth of the light and produces scattering due to differences in refractive indices of various tissue components. Materials with similar refractive index render similar OCT images and, therefore, it becomes difficult to identify those structures on the image [29]. Second, OCT is usually not a dynamic diagnostic tool and provides information at one point in time. More research work is required to develop a dynamic investigational tool to evaluate structural changes over a longer period of time. Most OCT systems designed for commercial use are bulky and, thus, are unsuitable for intraoral use. Currently, the OCT systems designed for dental use can be used primarily to scan the front teeth only. Last, most of the available research data are based on ex vivo studies. The clinical uses of OCT systems are not standardized and there is limited evidence to prefer OCT over other diagnostic tools.
